# Supersaturated Isotretinoin: Scrutiny into Solid States Attributes

**DOI:** 10.3390/ph19030430

**Published:** 2026-03-06

**Authors:** Rana Sejare, Sze Hui Ooi, Xin Yi Teoh, Ahmed Bassam Farhan, Siok Yee Chan

**Affiliations:** 1 School of Pharmaceutical Sciences, Universiti Sains Malaysia, Pulau Penang 11800, Malaysia; rana_sejare@hotmail.com (R.S.); ooiszehui@gmail.com (S.H.O.); 2 School of Pharmacy, Monash University Malaysia, Subang Jaya 47500, Selangor Darul Ehsan, Malaysia; txy1807@gmail.com; 3 Department of Pharmaceutics, College of Pharmacy, Baghdad University, Baghdad 10071, Iraq

**Keywords:** characterisation, polymorph, degradation, solid dispersion

## Abstract

**Background/Objectives**: The formulation development of Isotretinoin (ISN) is limited by its solubility and stability issues. This study aimed to characterise the BCS class II drug ISN, particularly the possible different solid state and formulate amorphous solid dispersion aiming for a supersaturation state. **Methods**: ISN’s physical states are investigated in its raw form, quench-cooled form, physical mixture with the polymer and corresponding solid dispersion form. Quench-cooled ISN was prepared in situ using DSC. Carrier stabilisation of ISN was attempted using the solid dispersion technique. Hereby, the solid dispersion of drug-polymer PVPVA at a ratio of 1:3 was prepared using the solvent evaporation method. Solid dispersion, physical mixture and raw ISN were characterised for the saturated solubility. Physical characterisation of the samples was performed using DSC, ATR-FTIR and a light microscope. **Results**: Two polymorphs of ISN (forms I and II) were found in the raw ISN, with form II being thermodynamically more stable. ISN possesses strong crystallinity and resistance to amorphisation under the applied quench-cooling condition without the presence of a carrier system. The conjugated polyene structure in ISN contributes to the polymorphic transformation and isomerisation. The incorporation of PVPVA in the solid dispersion system successfully improved the water solubility (sixfold) of ISN despite a combination of crystalline and amorphous components being present in the system. **Conclusions**: ISN is a class II drug crystal molecule. Taking advantage of solubility and possibility in the polymorphic transformation of ISN in a binary system, we concluded that ISN could potentially be formulated into its corresponding crystalline solid dispersion form.

## 1. Introduction

Isotretinoin (ISN) or 13-cis retinoic acid is a retinoid derivative of vitamin A used for the treatment of severe recalcitrant acne. ISN produces its effects by altering progress through the cell cycle, cell differentiation, survival and apoptosis [1,2]. The drug was approved by the U.S. Food and Drug Administration [3,4] and by European regulatory authorities (except Sweden) in 1982 and 1983, respectively [5], to treat severe, resistant, nodular acne that is unresponsive to conventional therapy, including systemic antibiotics [6]. Even after four decades, it continues to be the most effective acne treatment [7,8,9] and helps in acne scar prevention. Despite the clinical benefits of ISN, its bioavailability is limited to approximately 25% [3] in commercially available capsule formulations [10], primarily due to its low aqueous solubility and high lipophilicity [11]. ISN is sensitive to light, oxygen and heat and easily isomerises to tretinoin, which is a much higher placental transfer agent and a proven direct-acting dysmorphogen [12,13].

**Figure 1:**
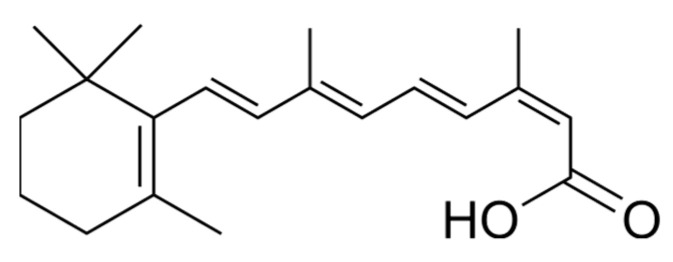
Molecular structure of ISN.

From the physicochemical perspective, ISN with a pKa of ~4 [14], Log P 5.66 [15], is soluble in chloroform, sparingly soluble in alcohol, isopropyl alcohol and polyethylene glycol 400 and practically insoluble in water [16]. Based on the study conducted by Shakeel, ISN mole fraction solubility was enhanced with an increase in temperature and the order in which it was soluble in the several solvents at 318.2 K, including ethyl alcohol (${3.51\times 10}^{-3})$ > ethane-1,2-diol ($3.49\times 10^{-3})$ > methyl-alcohol ($2.10\times 10^{-3})$ > water ($1.38\times 10^{-6}$) [17]. Figure 1 shows the molecular structure and physicochemical properties of ISN.

Structurally, ISN is a highly conjugated molecule consisting of a hydrophobic unit, which is the substituted cyclohexene moiety, and a nine-carbon polyene side chain with a terminal polar group, a carboxy group [18]. All but one of the double bonds $\left(C_{13}\right)$ in the side chain are trans, and it is the stereospecific construction of this polyene side chain [19,20]. Due to its long electron-rich conjugated polyene chain structure, ISN is easily influenced by various factors, including light with a wavelength less than 500 nm, oxygen, trace metals, strong acids and relatively high temperatures [21,22]. Based on reports of the solvent effect on the effective interaction of ISN, the carboxyl active group in ISN increases steric hindrance and reduces the hydrogen bond acceptor ability of this molecule via the carboxyl group [23]. Hence, the reorientation trend of the solvent molecules surrounding ISN molecules decreases. The bond lengths and angles of ISN show minor variations across solvents, indicating that the molecular framework is pretty robust but still responsive to solvent environments [23]. Additionally, polar solvents like water and dimethyl sulfoxide cause noticeable alterations, particularly in the carboxylic group region, which impacts the molecule’s interaction potential [24].

On 28 March 2024, Teva Pharmaceuticals USA, Inc initiated a nationwide recall of ISN Capsule USP 40 mg (lot number: 100044259). The recall was issued at the retail level because the 3-month stability result for the assay was found to be above the specification limit for some capsules of the specified lot [25]. This recall highlights the importance of understanding ISN’s physical and chemical stability in ensuring medication safety after marketing.

### Polymorphs of ISN

**Figure 2:**
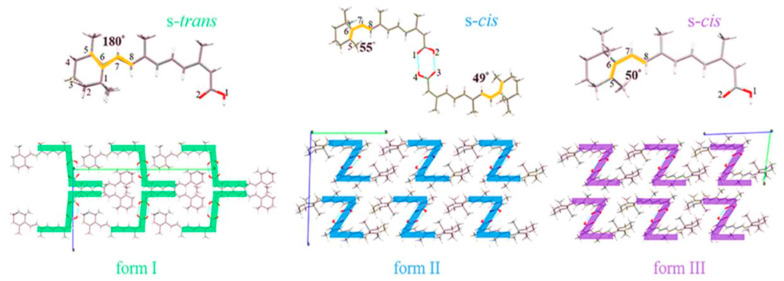
Crystal structures of three forms of ISN (Adapted from Cheng et al. 2021) [26].

There are three solid forms (form I, II and III) of ISN that have been reported up to date. The most remarkable variation between these polymorphs lies in the torsion angle between the $C_{5}$–$C_{6}$ of the trimethylcyclohexenyl ring and the $C_{7}$–$C_{8}$ of the polyene side chain. The apparent difference in colour for forms I, II and III could be explained by the different degrees of π-conjugation, as shown in Figure 2 [26]. Crystal form I (ϕ=180°), with the highest conjugation efficiency, presents an orange colour. Crystals of form II (ϕ=49° and 55°) or III (ϕ=50°) with less conjugation efficiency show a shorter wavelength colour of yellow.

Compared to the marketed form (Accutane^®^ manufactured by Roche as a reference product) with s-trans conformation, form II with s-cis conformation displays superior thermal stability and higher solubility at physiological temperatures [26], which leads to higher bioavailability. Therefore, form II holds broad application potential and may serve as a preferable alternative to the form of ISN available in the market.

The above-mentioned problems with ISN, including solubility and stability issues, necessitate the development of more stable and water-soluble solid forms. The strategies reported so far to mitigate the issues mainly include lipid-based formulations, particle size reduction and amorphous solid dispersion [27].

On the other hand, it was known that the existence of an amorphous state enhances the aqueous solubility of API due to the lack of a crystal lattice and the absence of strong lattice energy within the structure [28,29,30]. It was more interesting to postulate that amorphisation could tackle the poor aqueous solubility issue in ISN which directly affects its bioavailability. However, it is uncertain that ISN can exist in an amorphous state as no neat amorphous ISN has been reported. While the decision to formulate ISN into a crystalline or amorphous state remains debatable, understanding the solid states of ISN is crucial for initial screening to determine the ideal formulation design. Therefore, the aim of this study is to investigate and characterise the solid-state properties of ISN in support of formulation design decisions, by evaluating its physical state, crystalline characteristics and compatibility with the polymer. In this case, polyvinylpyrrolidone vinyl acetate (PVPVA) was chosen to be the potential carrier system in view of its hydrophilic nature, it being less hygroscopic and its ease of handling in the production compared to other carriers that have been used in a solid dispersion strategy.

## 2. Results

### 2.1. Differential Scanning Calorimetry

**Figure 3:**
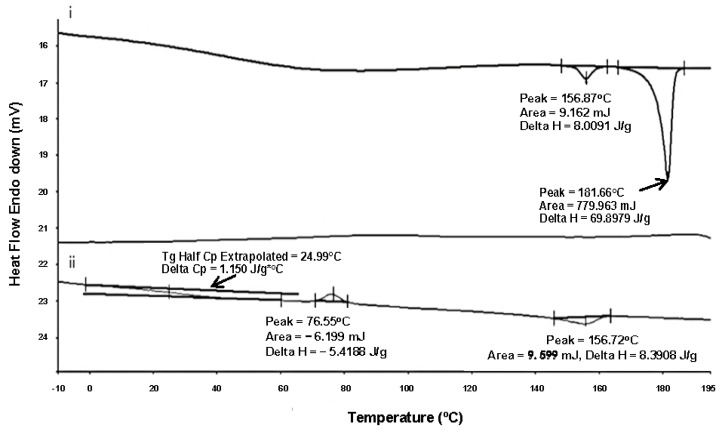
DSC thermograms of raw ISN (form I and II): (i) first heating cycle and (ii) second heating cycle.

The differential Scanning Calorimetry (DSC) results of raw ISN, as shown in Figure 3, show the existence of two polymorphs of ISN, form II and form I, which formed at 155 °C and 181 °C, respectively. The melting peaks of form I and form II ISN were consistent with a previous study by Cheng et al., 2021 [26].

During the first heating cycle, an endothermic peak was present at 155.87 °C (∆H=8.0091 J/g), corresponding to the melting point of form II ISN. Form I presented a significant endothermic peak at 181.66 °C (∆H=69.8979 J/g). During the second heating cycle, an exothermic peak was obtained at 76.55 °C (∆H=−5.4188 J/g), indicating recrystallisation of ISN upon cooling. This was then followed by an endothermic peak at 155.72 °C (∆H=9.599 J/g) corresponding to the melting point of form II ISN.

Gibbs free energy, ∆G, also known as free enthalpy, is the maximum energy which may be obtained from a system at a constant temperature and pressure [31]. Gibbs free energy provides chemical insights at the atomistic level by calculating the relative energies of the molecular crystals, taking into account the entropy and temperature effects of these molecular crystals [32,33]. The calorimetry data are useful in estimating the relative physical stabilities of crystalline polymorphs. At the melting point, the change in free energy from solid to liquid is zero, hence

(1) $$∆S_{m}=\frac{∆H_{m}}{T_{m}}\;$$

From the $T_{m}$ and $∆H_{m}\;$ data collected, $∆S_{m}$ can be calculated for each polymorph. Assuming that these parameters are approximately independent of temperature, for each polymorph, the relationship among $∆G_{m}$, $∆H_{m}$ and $∆S_{m}$ can be expressed as a function of temperature. According to the definition of the Gibbs energy function,

(2) $$∆G_{m}\;=\;∆H_{m}-T∆S_{m}$$

where G is Gibbs energy; H is enthalpy; T is absolute temperature; and S is entropy [34,35].

Polymorph structural stability is correlated with free energy, with the most stable polymorph having the lowest free energy [36]. Therefore, form I ISN is structurally more stable than form II based on the Gibbs free energy calculation, as shown in Table 1.

**Figure 4:**
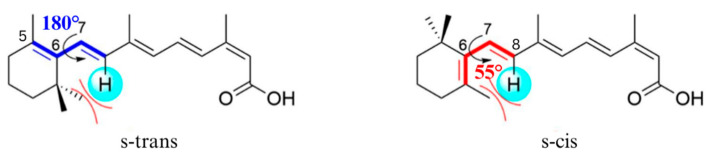
S-trans and s-cis conformers of ISN (Adapted from Cheng et al., 2021) [26].

However, the melting point of form I polymorph was absent in the second heating cycle, indicating the polymorphic transformation of form I polymorph upon the heating and cooling cycle. A similar observation has been observed with paracetamol, where polymorphic transformation causes favourable transformation into form II crystal after the heat–cool cycle, which is a metastable polymorph [37]. This could be further confirmed with Gibbs free energy, where form II ISN is considered a metastable polymorph. The different conformational structures, such as torsion angles [38], may explain the stability differences between the ISN polymorphs. The torsion angle between $C_{5}-C_{6}$ and $C_{7}-C_{8}$ of form I (Φ =180°) has s-trans conformation and form II (Φ =49° and 55°) has s-cis conformation [26]. This indicates that form I possesses a higher degree of π-conjugation [26,39]. Figure 4 demonstrates the s-trans and s-cis conformers of ISN. It was reported that a higher conjugation degree is beneficial to the stability of the intermediate, as it could react with the nearby molecules through conjugation [26,39].

**Figure 5:**
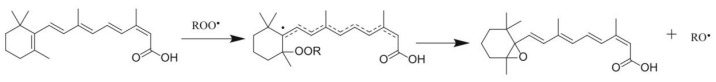
Possible oxidation mechanism of ISN under the action of heat and air (adapted from Cheng et al., 2021) [26].

ISN itself is sensitive to heat, and exposure to high temperatures Φ >200° [40] may cause the degradation of ISN, as shown in Figure 5. Hence, exposure to high temperatures should be avoided during production, storage and transportation [41].

#### 2.1.1. Quench-Cooled ISN

**Figure 6:**
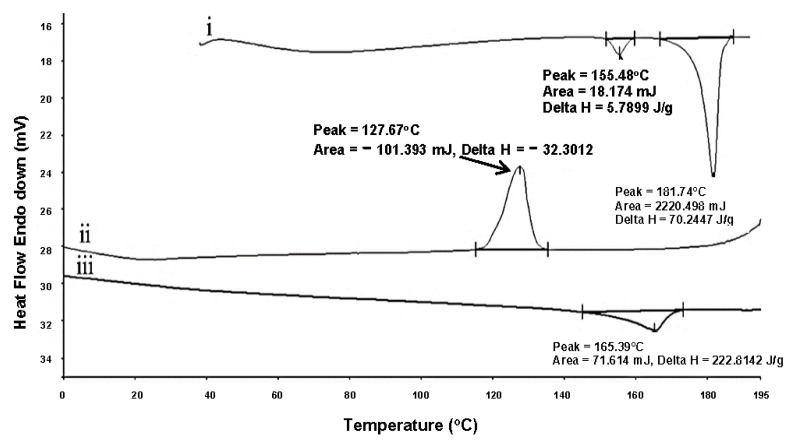
DSC thermogram of quench-cooled ISN: (i) first heating cycle, (ii) cooling cycle and (iii) second heating cycle.

The degree of crystallisation depends not only on the nature of the drug but also on the processing conditions, such as the cooling rate [42]. In our study, attempts at quench-cooling using DSC were carried out to detect the possibility of obtaining an amorphous state of ISN. Despite the higher cooling rate of 20 °C/min, recrytallisation of ISN occurred earlier, which was at 127.67 °C during the cooling cycle. During the second heating cycle, an endothermic peak was present at 165.39 °C. Theoretically, with an increasing cooling rate, the time for ISN to recrystallise will be shorter. This is because there is not enough time to rearrange in order to build crystals and thus the crystallinity will decrease accordingly [43]. Unlike the condition in Figure 3, the quench-cooled ISN did not undergo a cooling scan first; it retained a higher molecular mobility, allowing for rapid reorganisation into a crystalline form. The quench-cooled ISN profile is presented in Figure 6. Based on the result obtained, recrystallisation was noted at 127.67 °C in the quench-cooling cycle. This suggests that ISN possesses strong crystallinity and has high resistance to amorphisation under the applied quench-cooling conditions without the presence of a carrier system. Based on the behaviour of ISN during the DSC heating and cooling cycle, ISN is classified as class II molecules according to the classification reported in Baird et al., 2010 [44].

#### 2.1.2. DSC of ISN-PVPVA Solid Dispersion

**Figure 7:**
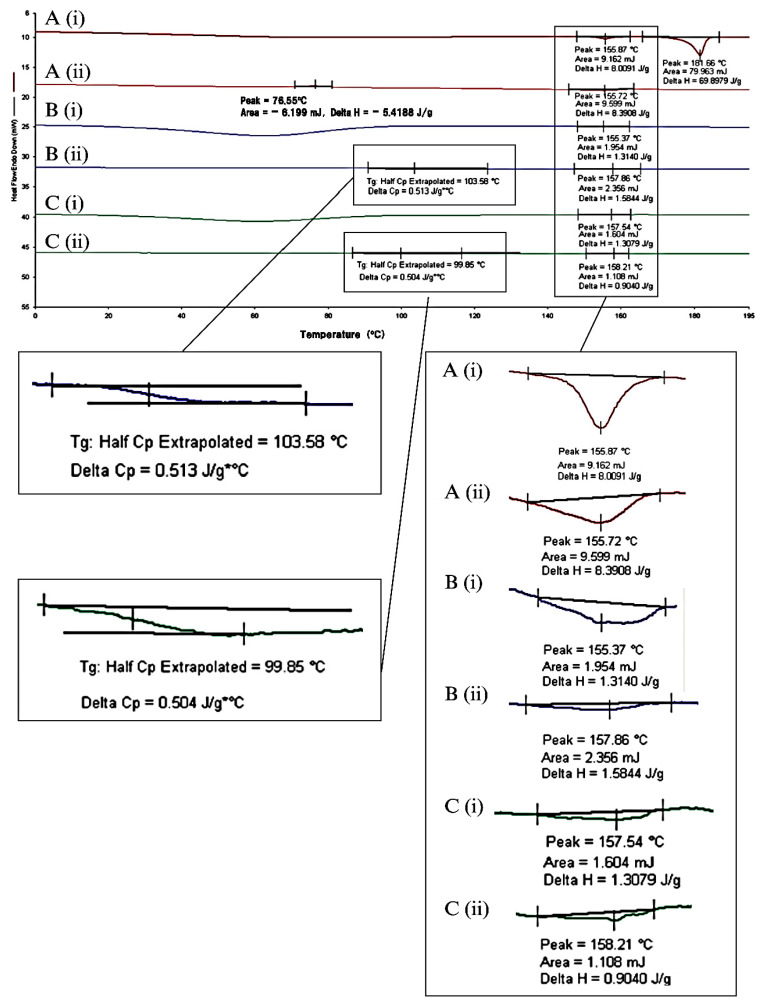
DSC thermogram of ISN samples: (A(i)) first heating cycle of raw ISN, (A(ii)) second heating cycle, (B(i)) first heating cycle of ISN-PVPVA PM, (B(ii)) second heating cycle of ISN-PVPVA PM, (C(i)) first heating cycle of ISN-PVPVA SD and (C(ii)) second heating cycle of ISN-PVPVA SD.

To stabilise the ISN molecules in their possible amorphous form, the addition of a carrier system is indispensable. The DSC results for different solid dispersion preparations of ISN with a heating rate of 10 °C/min are shown in Figure 7. There was a plausible $T_{g\;}$ detected at circa 99.85 °C, ISN-PVPVA SD also showed a melting endotherm at circa 158 °C  due to its partly crystalline property. This suggested the combination of amorphous and crystalline components present in the ISN-PVPVA SD system.

#### 2.1.3. Percentage of Crystallinity of ISN

The melting enthalpy, ∆$H_{PM}$, of PM and SD measured by DSC could be used to estimate the percentage of crystallinity of ISN that remained in the mixture. The crystallinity could be calculated using Equations (3) and (4).

(3) $$\%\;\mathrm{Crystallinity}=\frac{∆H_{{API}_{corrected\;in\;binary\;system}}}{∆H_{pure\;API}}\times 100$$

(4) $$∆H_{{API}_{corrected\;in\;binary\;system}}=\frac{∆H_{\mathrm{binary}\;\mathrm{system}}\times m_{\mathrm{binary}\;\mathrm{system}}}{m_{API\;in\;\mathrm{binary}\;\mathrm{system}}}$$

where $∆H_{pure\;API}$ is the enthalpy of pure API, $∆H_{PM}$ is the enthalpy attributed by the API in the binary system, $m_{binary\;system}$ is the total weight of the binary system and $m_{API\;in\;binary\;system}$ is the weight of API in the binary system. The degree of crystallinity calculated from DSC measurements is listed in Table 2.

According to Table 2, the physical mixture of ISN revealed less than 100% of crystallinity despite the simple mixing process using a mortar and pestle, which contrasts with the usual expectation. However, this observation was reported before due to the phenomenon of drug micro-dissolution in situ of the aluminium pan upon heating in DSC [45]. There is also a possibility that a certain portion of the crystalline ISN has turned amorphous through simple trituration due to mechanical intervention [46].

#### 2.1.4. *T_g_* Estimation via Gordon-Taylor Equation

The theoretical $T_{g\;}$ of the binary system was calculated using the Gordan–Taylor equation in Equation (5) based on the $T_{g\;}$ of single material as obtained in the DSC result.

(5) $$Tg=\frac{W_{1}{Tg}_{1}+KW_{2}{Tg}_{2}}{W_{1}+KW_{2}}$$

where $W_{1}$ and $W_{2}$ are the weight fractions of ISN and PVPVA, and ${Tg}_{1}\;$ and ${Tg}_{2}$ are the glass transition temperatures of ISN and PVPVA, respectively [47]. Table 3 lists all the numeric values used to calculate the theoretical $T_{g\;}$ and the estimated $T_{g\;}$ of all the investigated SD systems.

Based on the calculated $T_{g\;}$ the detected $T_{g\;}$ at 99.85 °C (Figure 7C(ii)) was higher than the theoretically predicted values, as listed in Table 3. This may indicate non-ideal mixing of ISN and PVPVA, whereby the polymer–drug interaction is positively deviated from ideal mixing, which signifies the comparatively lesser interaction between the two components in comparison to their corresponding pure forms.

### 2.2. Attenuated Total Reflectance Fourier Transform Infrared Spectroscopy (ATR-FTIR)

**Figure 8:**
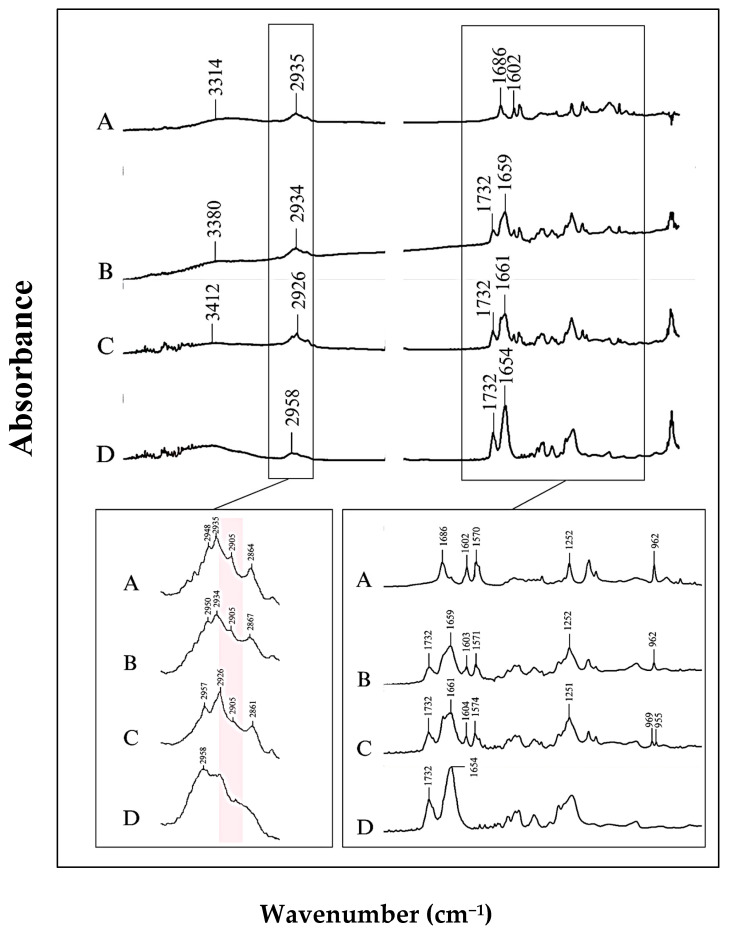
FTIR spectra of (A) raw ISN, (B) ISN-PVPVA PM, (C) ISN-PVPVA SD and (D) PVPVA. Pink region indicates the peak related to C-H bond in form I crystal.

ATR-FTIR was used to elucidate the mechanism of interaction between ISN and PVPVA which is necessary for drug–polymer miscibility. Figure 8 presents the FTIR spectra of the three prepared samples and PVPVA.

The samples showed the presence of important characteristic peaks (−OH, −CH, C=C, C−C, C=O), with some alterations in wavenumbers in comparison to pure ISN. The major peaks from the FTIR spectra of raw ISN at the wave number $3317\;{cm}^{-1}$ might be due to the stretching vibration of the −OH group and showed a strong peak. The strong peak at $2935\;{cm}^{-1}$ was due to the −CH stretch alkene, the strong peak at $1686\;{cm}^{-1}$ was due to the C=O group, the strong peak at $1602\;{cm}^{-1}$ was due to the C=C aliphatic group and the strong peak at $1570\;{cm}^{-1}$ was due to the C=C  aromatic group. The C−C aromatic group showed a strong peak at $1252\;{cm}^{-1}$, while the C=C aromatic group showed a strong peak at $962\;{cm}^{-1}$.

From the FTIR spectra of the ISN physical mixture and solid dispersion samples, it was observed that ISN and PVPVA were compatible with each other and there was no chemical reaction between them. PVPVA has a band at $1732\;{cm}^{-1}$, which is presented in the FTIR spectra of both the physical mixture and solid dispersion samples. There was no appearance of new bands in the FTIR spectra of the ISN physical mixture and solid dispersion samples, which strongly indicated no change in the drug structure.

There was broadening in the −OH stretching band of ISN molecules in solid dispersion preparation. It has also been noted that the C=O stretching in the physical mixture and solid dispersion was shifted to a lower frequency of $1659\;{cm}^{-1}$ and $1661\;{cm}^{-1}$, respectively, when compared to the spectra of the raw ISN sample ($1686\;{cm}^{-1})$. The carbocyclic acid, COOH group in ISN was believed to participate in the hydrogen bond interaction with PVPVA of the solid dispersion system. The characteristic peaks of raw ISN were consistent with the study by Nikam [47].

In the fingerprint region around $962\;{cm}^{-1}$, a single peak was observed for raw ISN and the physical mixture sample. However, a duplet peak was seen in the solid dispersion sample at 969 ${cm}^{-1}$ and 955 ${cm}^{-1}$. The peak at 962 ${cm}^{-1}$ corresponded to C−C  aliphatic stretching. In the physical mixture, ISN and PVPVA coexisted without interaction at the molecular level. Hence, ISN retained its original spectral signature. The appearance of the doublet peak in the solid dispersion sample at a region of 969 cm^−1^ was different from the raw ISN, which may suggest molecular interactions between the drug and polymer after the process of solvent evaporation. Here, the duplet is postulated as an indicator of a shift in the molecular environment due to the interaction between ISN and functional groups in PVPVA through hydrogen bonding or dipole–dipole interaction. This interaction may lead to slight differences in vibrational energy as the ISN is no longer in a homogeneous environment.

**Figure 9:**
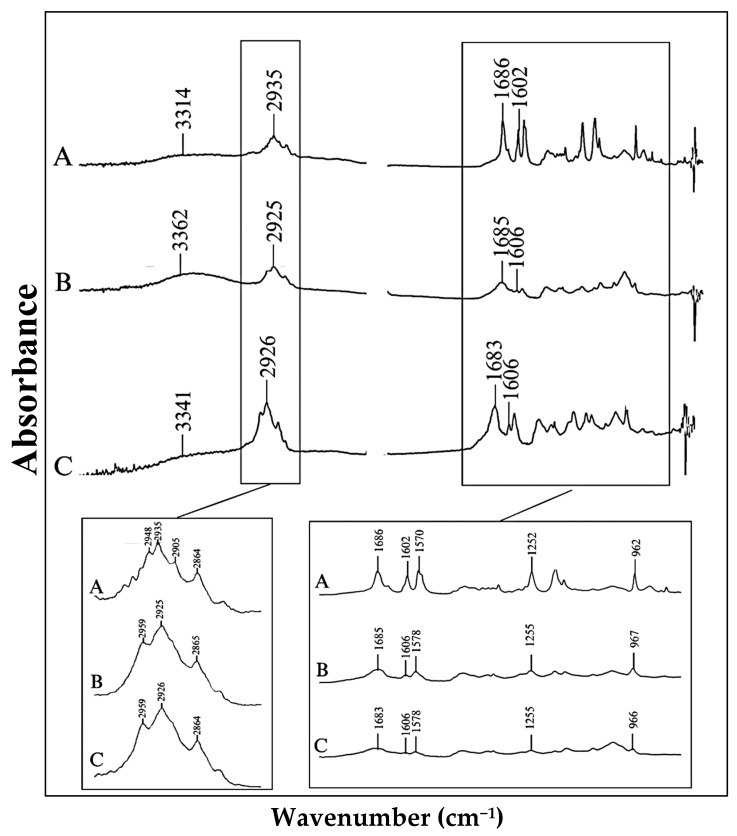
FTIR spectra of (A) raw ISN, (B) DSC-treated raw ISN and (C) DSC-treated quench-cooled ISN.

Another interesting observation was found at the region around $2850-2960\;{cm}^{-1}$, which corresponded to C−H stretching vibration of the alkene. In the raw ISN, this region displayed a quartet pattern, with sharp peak at 2905 ${cm}^{-1}$, whereas the physical mixture and solid dispersion ISN samples showed a broadening peak at 2905 ${cm}^{-1}$. The broadening peak might be due to weakening of the C−H bond in form I crystal after undergoing the process of mixing and solvent evaporation. Similar findings were reported for the form I and II polymorphs of paracetamol [48]. Therefore, it can be proposed that the significant peak patterns in this region can be used as a potential indicator to distinguish features between the polymorphs of ISN. To further support this hypothesis, raw ISN samples treated by DSC, which only contain a form II polymorph, were analysed under FTIR. The FTIR spectra of the samples are presented in Figure 9. These treated samples displayed triplet peaks in the same region ($2850-2960\;{cm}^{-1})$, confirming the absence of a peak at 2905 ${cm}^{-1}\;$ and reinforcing the potential of the C−H stretching region as a spectral marker for differentiating ISN polymorphs.

### 2.3. Light Microscopy

Light microscopy was used to image the morphology of ISN samples before and after the solvent evaporation processing. Polarised light was utilised to determine the possible crystallinity conversion of ISN in the solid dispersion samples. The presence of birefringence indicates the presence of crystalline content in the sample.

The raw ISN particles appeared as elongated, needle-like crystals with sharp edges. On the other hand, the physical mixture of ISN and PVPVA was significantly smaller and more irregularly shaped as compared to raw ISN, appearing as small granules. This size reduction might be attributed to the trituration process using a mortar and pestle. The solid dispersion ISN/PVPVA was larger and irregularly shaped and appeared more plate-like. During the preparation of solid dispersion through solvent evaporation, rapid removal of the solvent by vacuum drying [49] might lead to uncontrolled precipitation and particle aggregation. When the dried product was removed from the rotatory evaporator flask, it formed larger, irregularly shaped particles due to the adherence of the material to the flask’s surface and subsequent mechanical scraping. This process could result in uneven particle sizes and morphology, which may impact the dissolution rate and overall physicochemical properties of the formulation.

**Figure 10:**
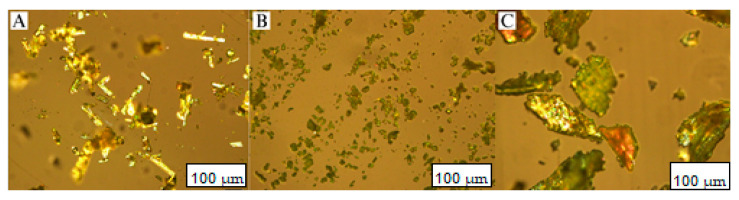
Morphology of (**A**) raw ISN, (**B**) ISN-PVPVA PM and (**C**) ISN-PVPVA SD under polarised light microscopy at 10× magnification.

As shown in Figure 10, birefringence was observed from the raw ISN, indicating the crystalline properties of ISN, in agreement with the analyses in the DSC.

On the other hand, the ISN physical mixture and solid dispersion samples also showed a lower intensity of birefringence, which implies the reduction in molecular ordering. This is consistent with the finding of DSC, where minute crystalline materials have been detected in PM and SD samples because melting enthalpy of the ISN was detected, but to a lesser extent compared with raw ISN. The reduced crystallinity of the SD in this study has indicated the capability of PVPVA in maintaining a certain portion of amorphous ISN, but not completely.

### 2.4. Saturated Solubility

Saturated solubility tests were conducted for raw ISN, ISN-PVPVA PM and ISN-PVPVA SD samples in distilled water at 25 °C for 24 h. The results of the saturated solubility of ISN were found to be within the range of the calibration curve between 0.5 and 12 µg/mL. As can be seen in Table 4, the solid dispersion sample showed a significant improvement in its apparent solubility.

The incorporation of polymer PVPVA may help in stabilising the chemical structure of ISN. When physically mixed with PVPVA, the solubility of ISN increased slightly to  3.845 μg/mL, demonstrating a mild enhancement in the solubility. This might be due to a decrease in the particle size of ISN when mixed with PVPVA in a mortar and pestle. Considering that PVPVA is a strong hydrophilic carrier, it would increase the wettability of ISN, thereby improving its apparent solubility. The observed apparent solubility enhancement could also be ascribed to the drug polymer interaction, which has been shown in the IR spectra.

## 3. Discussion

ISN is a very lipophilic molecule, but the conjugated polyene side chain in the molecule structure was labile to heat, light and air. These physicochemical properties impose considerable limitations on therapeutic formulation development [50]. Hence, its application was limited by stability issues and low solubility.

For drugs that crystallise rapidly such as ISN, crystalline solid dispersion has a significant advantage over amorphous solid dispersion due to the reduced crystallisation tendency [51] and relatively stable molecular mobility [52,53]. In this study, the ratio of ISN-PVPVA 1:3 was chosen based upon the theoretical estimation of 1-to-3 drug polymer interaction that has been previously reported by Chan et al. 2015a and Chan et al. 2015b to be one of the determining factors in SD stabilisation [45,54]. The results of the saturated solubility of ISN-PVPVA SD were remarkably promising in stabilising the drug and tackling the issue of the poorly soluble nature of ISN in its neat form. This improvement is postulated to contribute positively to the dissolution performance of the drug in its solid dispersion form. Intermolecular interactions are believed to affect the drug solubility by altering the drug crystallite size [55]. The interaction between ISN and PVPVA mainly occurs through hydrogen bonding [56] which is supported by the ATR-FTIR findings. When drugs are dispersed in polymers, interactions such as hydrogen bonding between ISN and PVPVA influence the molecular mobility of ISN [57], thereby altering its crystallisation rates [58,59].

In general, rapid cooling or solvent evaporation results in polymorphic transformation in the process of precipitation, which typically promotes the formation of metastable polymorphs [60]. In the current case, form II, the metastable form of ISN, was obtained in the presence of PVPVA through the solvent evaporation method with apparent reduction in crystallinity based upon the result of melting enthalpy in the thermogram. With the further reduction in DSC melting enthalpy found in SD samples, it could be deduced that there is a portion of ISN that has been transformed into its corresponding amorphous counterpart stabilised in the presence of PVPVA. This could be attributed to the drug polymer interaction that assists in drug stabilisation. It has been reported that a different SD preparation method may also contribute to the different thermal history of the mixture and result in different degrees of amorphisation and polymorphic transformation. Citing the theoretical calculation of molar ratio between the drug and polymer, where ISN has one proton donor and PVPVA possesses two proton acceptors, a hydrogen bond was shown in the IR spectra and preferably interacted at pyrrolidone carbonyl, which is in agreement with previously reported work [54]. With the interesting finding of ATR-FTIR at the region of circa 2900 cm^−1^, the ISN was found to interact differently in quench-cooled and DSC-treated samples. These spectra resemble the one seen in SD, samples indicating presence of form II ISN. Thus, if the intention is to prepare a solid dispersion containing ISN form I, this solvent evaporation method might not be suitable. Drug–polymer interaction has been suggested to be important in physical stabilisation [54]. This may be a contributing factor that prevents the cis-trans isomerisation of ISN to tretinoin in the current study.

To recap, solubility enhancement of ISN was noted in the presence of PVPVA (Table 4). It is worth mentioning at this juncture that, the solubility test of the raw ISN (without PVPVA) was found to reveal Tretinoin’s UV spectrum detected at 220 nm. The absorbance peak at 220 nm indicated the presence of a conjugated polyene structure and cis-trans transformation [61]. A previous report has declared the ISN-tretinoin interconversion problem in methanolic solution when exposed to UVA and visible light [23,62,63]. However, this is not seen in the current calibration curve presented in Section 4.3. It was found that the sequences of the mixing process lead to different conversion outcomes. Namely, the solubility test of the ISN in pure distilled water overnight causes interconversion (beaker was wrapped with aluminium foil), but a mixture of 50:50 ethanol water did not escalate the conversion issue. The addition of ethanol in the drug before distilled water has protected ISN from undergoing conversion to tretinoin in a short time frame. This conversion process has also been reported to be facilitated by photoisomerisation and photolysis upon light exposure [23] through changes in their configuration of double bonds, as the conjugated polyene systems are highly sensitive to light. The problem of ISN–tretinoin interconversion was, however, not detected in the physical mixture and solid dispersion samples, which signifies the important of the solid dispersion strategy in maintaining the ISN nature, a less toxic version compared to the tretinoin.

## 4. Materials and Methods

### 4.1. Materials

PVPVA was purchased from BASF to be used as a carrier in the solid dispersion system of ISN, while Isotretinoin (ISN) was supplied by Wuhan Senwayer Century Chemical Co., Ltd. (Wuhan, China).

### 4.2. Preparation of ISN Physical Mixture (PM)

PM of ISN and PVPVA at a ratio of 1:3 was prepared by trituration mixing of the weighed powders using a pestle and mortar. This ratio of API-PVPVA was reported by Chan et al. 2015b to be able to stabilise a rapidly recrystallising drug molecule, particularly a molecule with a hydrogen bond donor, as the hydrogen bond could be formed with the polyvinylpyrrodine carbonyl’s moiety [54].

### 4.3. UV-Vis Spectroscopy

**Figure 11:**
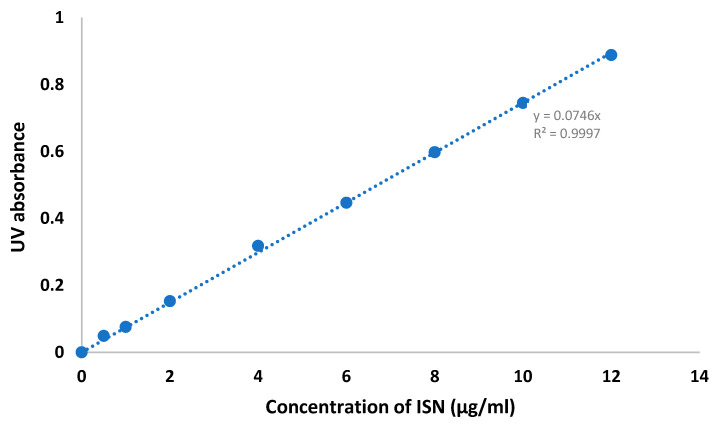
A representative calibration curve for ISN dissolved in water/ethanol 50/50 at 357 nm.

ISN was dissolved completely in ethanol via sonication in a Sonica^®^ ultrasonic bath (Milan, Italy) for 30 min to ensure complete solubilisation before the addition of distilled water to make up a stock solution of 25 µg/mL ISN in water/ethanol 50/50. The stock solution was further diluted with 50:50 ethanol:water to produce a linear calibration curve ranging from 0.5 to 12 µg/mL, R^2^ = 0.999 at the λmax of 357 nm using a UV-Visible Spectrophotometer Double Beam U2900 (Hitachi High-Tech, Tokyo, Japan). Figure 11 shows the standard calibration curve of ISN dissolved in water/ethanol 50/50.

### 4.4. Preparation of ISN Solid Dispersion (SD) Using the Rotary Evaporation Method

The amount of ISN and PVPVA at a ratio of 1:3 was weighed. The two aforementioned powders were mixed and dissolved in the ethanol solution with magnetic stirring at 300 rpm at 100 °C to form a clear solution. The solution was then subjected to solvent removal through co-evaporation under reduced pressure and elevated temperature using the Eyela N-1000 rotatory evaporator (EYELA, Tokyo, Japan). The solution was placed in a flask that was wrapped up with aluminium foil to prevent any chance of photodegradation of ISN during the preparation process; then, the flask rotated at a constant speed and at a temperature of 50 °C under reduced pressure (−600 mm Hg) [64,65]. This caused the liquid to evaporate, leaving behind the SD samples. After evaporation, the resulting products were allowed to cool and dry before being collected from the round-bottom flask. The condensed unwanted liquid was then discarded.

### 4.5. Physical Characterisation of Samples

Solid-state characterisation of the raw ISN, prepared PM and SD of ISN and PVPVA were carried out by scanning in differential scanning calorimetry (DSC) and attenuated total reflectance–Fourier transform infrared (ATR-FTIR). The samples were also tested under light microscopy. Saturated solubility was carried out for all samples. All examinations were conducted in triplicate.

#### 4.5.1. Differential Scanning Calorimetry

In this study, DSC analysis was performed using DSC (Perkin Elmer Pyris 6 DSC, Shelton, CT, USA). Thermal analysis was carried out by weighing 2–5 mg of each ISN sample in an aluminium pan and sealing it. The samples of ISN (raw, PM and SD) were heated from −20.00 °C to 200.00 °C at a constant scanning rate of 10.00 °C/min under a dry helium flow of 20 mL/min. An empty aluminium pan with an equivalent weight was used as a reference.

Attempts have been made to detect the possibility of obtaining an amorphous state of ISN by preparing quench-cooled ISN in situ in DSC [66]. The quench-cooled sample was prepared by weighing an accurate amount of raw ISN in an aluminium standard pan. Then, it was crimped and heat with a protocol from 40.00 °C to 195.00 °C at a constant scanning rate of 10.00 °C/min for the first heating cycle. After an isothermal of one minute inside the furnace of DSC, the pan was cooled to −20 °C at a cooling rate of 20.00 °C/min before the second heating cycle. After an isothermal for 1 min, the same sample was heated again from −20 °C to 200 °C at the third cycle. The results obtained were analysed using Pyris Data Analysis software (Version 9.1.0.0203; Perkin Elmer, 2009) to track the thermal characteristic changes of the compound.

#### 4.5.2. Attenuated Total Reflectance Fourier Transform Infrared Spectroscopy

Attenuated total reflectance–Fourier transform infrared (ATR FTIR) spectra were recorded over a wavenumber range of 600 cm^−1^ to 4000 cm^−1^ with a resolution of 4 cm^−1^ and 32 scans using a Thermo Nicolet FTIR Nexus spectrometer (Thermo Fisher Scientific, Madison, WI, USA) coupled with a Germanium ATR Crystal accessory. A sufficient weight (1–5 mg) of powdered ISN (raw, quench-cooled ISN in situ by DSC, PM and SD) was placed directly onto the ATR crystal to ensure full coverage and good contact [67]. The spectra obtained were analysed using OMNIC software (OMIC 9.2.86).

#### 4.5.3. Light Microscopy

The microscopic images of surface morphology for ISN samples (raw, PM and SD) were examined under the optical Olympus microscope (DP72 Microscope Digital Camera (Olympus Corporation, Tokyo, Japan) with a U-POT polariser at room temperature. The sample was placed on a microscope slide and then photographed using magnification of a 10× lens. The images were analysed by the cell Sens Standard software (Build 8624) of the Olympus corporation.

#### 4.5.4. Saturated Solubility

Saturated solutions of ISN (raw, PM and SD) were prepared by adding an excess amount, circa 20 mg of the sample in 10 mL of distilled water. The solution was stirred at 300 rpm for at least 24 h at room temperature and filtered through a 0.45 μm cellulose acetate membrane filter before UV spectroscopy analysis and appropriately diluted with the same volume of ethanol; then, it was scanned using a UV-Visible Spectrophotometer Double Beam U2900 between 200 nm to 400 nm on the spectrum mode to determine the transformation of the chemical structure of ISN samples. The absorbance of all samples at the $\lambda_{max}$ of 357 nm was recorded to calculate the saturated solubility. The blank was water/ethanol 50/50. three successive samples were taken after 24 h to ensure the amount of the drug detected indicated apparent solubility of the sample, which reached a plateau. The experiment was performed in triplicate.

### 4.6. Statistical Analysis

The results of the saturated solubility study were analysed using an independent-samples *t*-test. Values of *p* < 0.05 were considered statistically significant. The results are presented as the means ± SD.

## 5. Conclusions

The current work presents the investigation of the solid-state characteristics of a poorly water-soluble drug, Isotretinoin (ISN). ISN is a class II drug crystal molecule. Solid dispersion of drug-polymer PVPVA at a ratio of 1:3 was prepared using the solvent evaporation method. The solid dispersion, physical mixture and raw ISN were characterised for saturated solubility. It was demonstrated that raw ISN exists as a mixture of polymorphs form I and II, with form II being thermodynamically more stable under the current processing method. Attempts to obtain the amorphous state of ISN via quench-cooling using DSC have suggested that ISN possesses strong crystallinity and resistance to amorphisation in its pure form. Even though a neat amorphous form of ISN could not be obtained, it was found that there was a polymorphic transformation of ISN in the ISN-PVPVA physical mixture and possible conversion into partially amorphous states with a binary mixture of PVPVA. A further increment of saturated solubility (sixfold) of ISN in the ISN-PVPVA SD was observed and postulated to contribute to the dissolution performance of the drug. Taking advantage of solubility and possibility in the polymorphic transformation of ISN in a binary system, we concluded that ISN could potentially be formulated into its corresponding partially crystalline solid dispersion form.

## Figures and Tables

**Table 1:** Calculation of Gibbs energy of ISN form I and form II polymorphs at room temperature, T = 298.15 K.

Polymorphic Form of ISN	I	II
$∆H_{m}$ (J/g)	69.8979	8.0091
$T_{m}$ (°C)	181.66	155.87
$∆S_{m}$ (J g^−1^°C^−1^)	0.3848	0.0514
$∆G_{m}$ (J/g)	−44.83	−7.32

**Table 2:** Percentage of crystallinity of ISN in physical mixture and solid dispersion with PVPVA.

Binary Systems	Crystallinity Calculated from DSC Enthalpy (%)
ISN-PVPVA PM	75.54
ISN-PVPVA SD	43.09

**Table 3:** Values used to calculate theoretical $T_{g\;}$ based on the Gordon–Taylor equation.

Component	$∆{Cp}_{1}$ (J/g∗°C)	$∆{Cp}_{2}$ (J/g∗°C)	K( $∆{Cp}_{2}/∆{Cp}_{1})$	${Tg}_{1}$ (°C)	${Tg}_{2}$ (°C)	$\mathrm{Theoretical}\;T_{g}$ of ISN-PVPVA
PVPVA	-	0.510	-	-	104.4 ^#^	-
ISN	1.150	-	0.443	24.99 *	-	70.30

* Putative $T_{g\;}$ of ISN obtained from our current study based on Figure 3. ^#^ $T_{g\;}$ of PVPVA obtained from (Chan et al., 2015 a [45]).

**Table 4:** Analysis of independent samples *t*-test on the saturated solubility of ISN samples in distilled water at 25 °C, UV wavelength =357 nm, n=3.

Sample	Experimental Solubility (μg/mL)		
	Distilled Water	T Statistic (*df*)	*p*
Raw ISN	<LOD (0.5)	-	-
ISN-PVPVA PM	3.845 ± 0.0958	−93.33 (4)	<0.0001
ISN-PVPVA SD	23.056 ± 0.3433		

## Data Availability

The original contributions presented in this study are included in the article. Further inquiries can be directed to the corresponding author(s).

## Abbreviations

The following abbreviations are used in this manuscript:

ATR-FTIR	Attenuated total reflectance–Fourier transform infrared
DSC	Differential Scanning Calorimetry
ISN	Isotretinoin
PM	physical mixture
PVPVA	polyvinylpyrrolidone vinyl acetate
SD	solid dispersion
